# Latent Autoimmune Diabetes in Adults: Diagnostic and Therapeutic Challenges

**DOI:** 10.7759/cureus.92356

**Published:** 2025-09-15

**Authors:** Jimmy Joseph

**Affiliations:** 1 Internal Medicine, Aster DM Healthcare, Dubai, ARE

**Keywords:** anti-gad antibody, insulin, islet cell antibody, lada, type 1.5 diabetes

## Abstract

Latent autoimmune diabetes in adults (LADA) represents a distinct form of autoimmune diabetes that manifests in adulthood and is often misdiagnosed as type 2 diabetes mellitus (T2DM). It is characterized by autoimmune destruction of pancreatic β-cells, the presence of circulating autoantibodies, and a gradual but inevitable progression to insulin dependence. This hybrid phenotype combines features of both type 1 and type 2 diabetes, resulting in frequent diagnostic delays and suboptimal management.

We report the case of a 36-year-old non-obese male who initially presented with symptomatic hyperglycemia and was treated as T2DM with oral hypoglycemic agents. Despite adherence to metformin and glimepiride, his glycemic control worsened over the following months. Further evaluation revealed low C-peptide levels and positivity for anti-glutamic acid decarboxylase (GAD65) and islet cell antibodies (ICA), establishing a diagnosis of LADA. He was transitioned to basal-bolus insulin therapy and followed for 18 months, during which he achieved improved metabolic control with HbA1c reduction from 9.2% to 6.9%.

This case underscores the clinical importance of distinguishing LADA from classical T2DM. The presence of autoantibodies and low endogenous insulin reserve should alert clinicians to the possibility of autoimmune diabetes. Early insulin initiation is advocated to preserve residual β-cell function and minimize long-term complications. In addition, patient education and individualized therapy are crucial in optimizing outcomes.

Awareness of LADA among physicians is critical, especially in young and middle-aged adults with atypical features of T2DM, such as lean body habitus, rapid glycemic deterioration, or poor response to oral therapy. This case adds to the growing body of evidence supporting the role of antibody testing and C-peptide assessment in adult-onset diabetes, facilitating accurate classification and timely therapeutic intervention.

## Introduction

Latent autoimmune diabetes in adults (LADA) is a slowly progressive form of autoimmune diabetes characterized by the presence of islet autoantibodies, gradual β-cell decline, and clinical onset in adulthood [1]. Although initially classified under type 1 diabetes mellitus (T1DM), LADA exhibits clinical overlap with type 2 diabetes mellitus (T2DM), often leading to misclassification [2]. The Immunology of Diabetes Society (IDS) proposed criteria defining LADA as: (i) age of onset greater than 30 years, (ii) the presence of circulating islet autoantibodies, and (iii) absence of insulin requirement for at least six months after diagnosis [3].

The prevalence of LADA varies widely, ranging from 2% to 12% among patients initially diagnosed with T2DM, depending on ethnicity and population studied [4]. It is estimated that nearly 10% of presumed T2DM cases in European populations are actually LADA [5]. Patients typically present with hyperglycemia in early to middle adulthood, a lean or normal body mass index (BMI), and suboptimal response to oral hypoglycemic agents (OHAs) [6]. The natural course involves progressive β-cell destruction and eventual insulin dependence, usually within five years of diagnosis [7].

Several autoantibodies are associated with LADA, the most common being anti-glutamic acid decarboxylase (anti-GAD65), which is present in 70%-90% of cases [8]. Other markers include islet cell antibodies (ICA), insulinoma-associated antigen-2 antibodies (IA-2), and zinc transporter 8 antibodies (ZnT8) [9]. The presence and titers of these antibodies not only confirm the autoimmune basis but also correlate with the rate of β-cell decline [10].

Differentiating LADA from T2DM is of clinical significance, as management strategies differ. OHAs are often ineffective, and sulfonylureas may accelerate β-cell exhaustion [11]. Insulin therapy remains the mainstay, with emerging evidence suggesting that early initiation of insulin may help preserve residual β-cell function [12]. Adjunctive use of dipeptidyl peptidase-4 inhibitors, GLP-1 receptor agonists, and SGLT2 inhibitors is under investigation [13].

Misclassification of LADA as T2DM can result in delayed initiation of insulin therapy, poor glycemic control, and increased risk of complications such as retinopathy, nephropathy, and cardiovascular disease [14]. Hence, screening for autoantibodies in non-obese adults with poor response to oral therapy is recommended [15].

This case presents a 36-year-old male with autoimmune antibody positivity and progressive β-cell failure consistent with LADA. The case highlights diagnostic challenges, treatment decisions, and the importance of long-term follow-up in such patients.

## Case presentation

A 36-year-old male with no prior comorbidities presented with polyuria, polydipsia, fatigue, and unintentional weight loss of 5 kg over three months. He had no history of hypertension, dyslipidemia, or autoimmune disease. Family history was negative for type 1 diabetes. On examination, his BMI was 23.5 kg/m².

Initial laboratory evaluation showed fasting plasma glucose 178 mg/dL, 2-hour postprandial glucose 264 mg/dL, and HbA1c 8.4%. Urine albumin-to-creatinine ratio (UACR) was 18 mg/g (within normal limits). Lipid profile was normal (total cholesterol 172 mg/dL, LDL 92 mg/dL, HDL 48 mg/dL, triglycerides 120 mg/dL). Creatinine was 0.9 mg/dL, also within normal limits. Based on these results, he was diagnosed with type 2 diabetes mellitus. Lifestyle counseling on structured meals and physical activity was provided, and he was started on metformin 1000 mg twice daily and glimepiride 2 mg daily.

After three months, HbA1c worsened to 9.2% despite adherence. Repeat evaluation showed fasting C-peptide 0.4 ng/mL (low), anti-GAD65 antibodies 72 IU/mL (positive), and islet cell antibodies (ICA) positive. Thyroid function was normal, and thyroid peroxidase antibodies were negative. Based on antibody positivity and low C-peptide, the diagnosis was revised to LADA.

Oral agents were discontinued, and the patient was initiated on a basal-bolus insulin regimen with insulin glargine 18 units at bedtime and insulin aspart 6 units before meals. Metformin was continued for insulin sensitization. During 18 months of follow-up, his HbA1c and fasting glucose trends are shown in Table 1.

**Table 1:** Glycemic and HbA1c trends HbA1c: glycosylated hemoglobin, FPG: fasting plasma glucose.

Time (months)	HbA1c (%)	FPG (mg/dL)	Therapy Adjustments
0	8.4	178	Metformin + glimepiride
3	9.2	192	Switched to basal-bolus insulin
6	7.5	130	basal-bolus insulin
12	7.1	118	basal-bolus insulin
18	6.9	110	basal-bolus insulin

Therapeutic course

The oral hypoglycemic agents were discontinued, and the patient was transitioned to a structured basal-bolus insulin regimen to achieve optimal glycemic control. Basal coverage was provided with insulin glargine, initiated at 8 units administered at bedtime and progressively titrated over a three-month period to 20 units, with adjustments guided by fasting glucose measurements to consistently maintain values below 130 mg/dL. For prandial control, bolus therapy was introduced with insulin aspart, beginning at 6 units before each meal. Based on postprandial glucose monitoring, the dose was carefully escalated to 8 units with breakfast and lunch, and 10 units with dinner to counteract higher evening glycemic excursions. In addition to insulin therapy, metformin at a dose of 1000 mg twice daily was continued as an adjunct agent to enhance insulin sensitivity and improve metabolic outcomes. This comprehensive therapeutic approach allowed for individualized titration of insulin doses while ensuring continued benefit from metformin’s established role in type 2 diabetes management.

Follow-up course over 18 months

**Figure 1:**
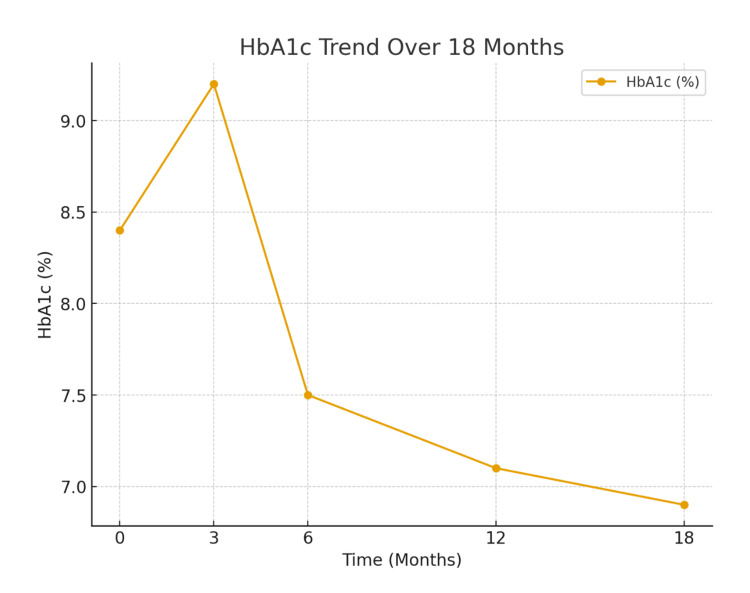
HbA1c trend in 18 months HbA1c: glycosylated hemoglobin.

**Figure 2:**
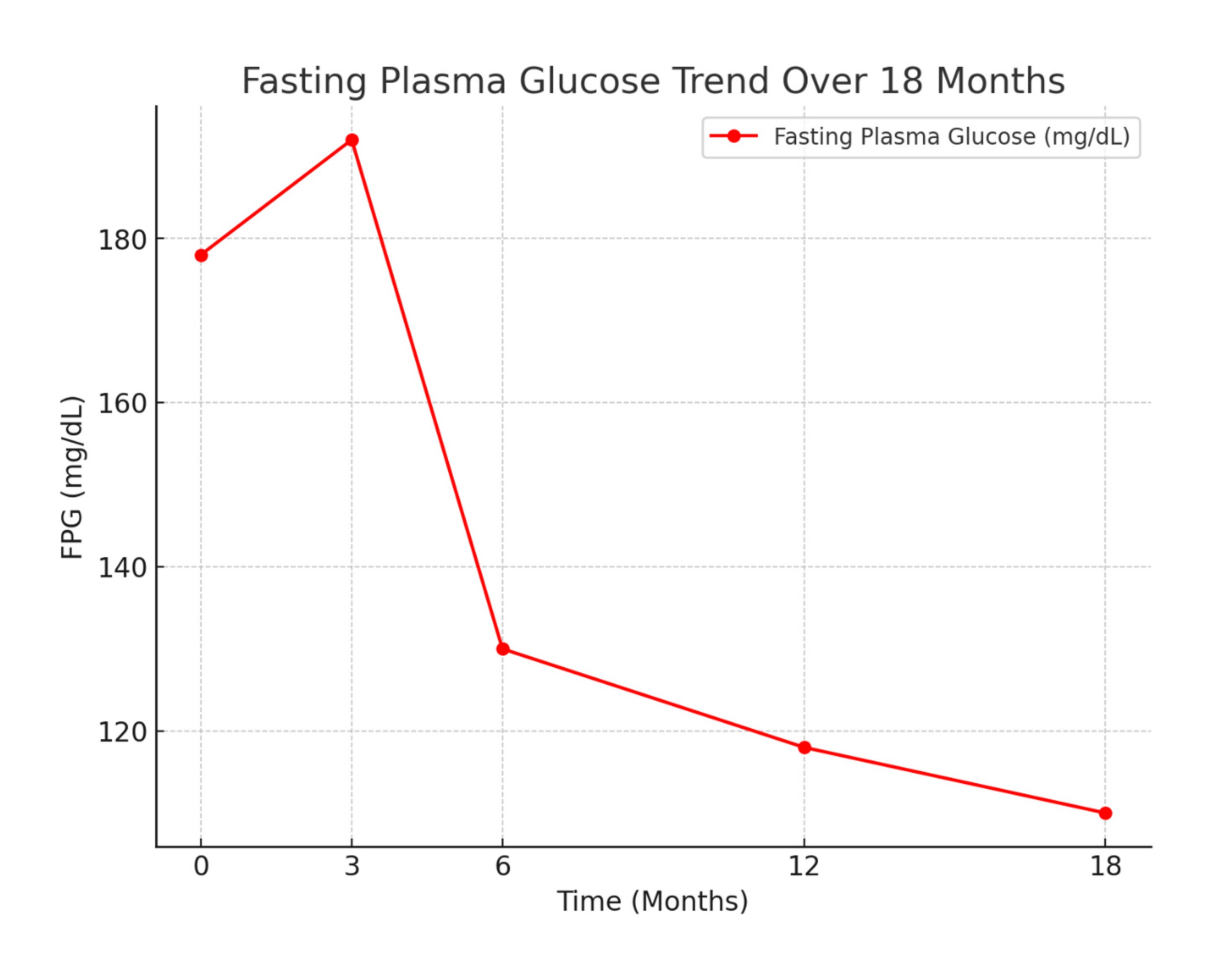
FPG trend in 18 months FPG: fasting plasma glucose.

Over 18 months, the patient demonstrated steady improvement in glycemic control with close monitoring and titration of therapy. At the 6-month follow-up, HbA1c had improved to 7.5%, with insulin glargine stabilized at 20 units nightly and insulin aspart adjusted to 8 units with breakfast, 8 units with lunch, and 10 units with dinner. By 12 months, HbA1c had further reduced to 7.1%, with no reported episodes of hypoglycemia. During this period, lifestyle counseling was reinforced, focusing on structured meal planning and incorporation of regular physical activity to complement pharmacologic therapy. At 18 months, HbA1c showed further improvement to 6.9% (Figure 1), and fasting glucose stabilized at 110 mg/dL (Figure 2). The patient remained on a stable regimen of insulin glargine 20 units at night and insulin aspart 8-8-10 units before meals, which continued to provide effective and well-tolerated glycemic control.

Throughout follow-up, UACR and lipid profile remained within normal limits. The patient demonstrated good adherence, tolerated therapy well, and reported improved quality of life.

## Discussion

LADA poses unique diagnostic and therapeutic challenges because it shares clinical features with both type 1 and type 2 diabetes [1,2]. This patient, with adult-onset diabetes, normal BMI, poor response to oral agents, and antibody positivity, fulfilled IDS criteria [3].

Management strategies differ significantly between LADA and type 2 diabetes. Sulfonylureas have been shown to accelerate β-cell decline [11], while early insulin therapy may preserve residual β-cell function and improve metabolic control [12]. In this case, insulin initiation resulted in sustained glycemic improvement and stabilization of fasting glucose. This finding aligns with previous long-term studies showing that early insulin therapy slows the deterioration of β-cell reserve [12].

The role of adjunctive agents remains under exploration. GLP-1 receptor agonists and DPP-4 inhibitors may offer β-cell preservation [13], though evidence is limited. SGLT2 inhibitors provide cardiovascular and renal benefits, but their role in LADA is not yet established and carries a potential risk of euglycemic diabetic ketoacidosis [14].

Screening for thyroid autoimmunity is also important, as autoimmune thyroiditis frequently coexists with LADA [9]. This patient tested negative for thyroid antibodies but warrants periodic reassessment.

This case emphasizes the clinical heterogeneity of LADA and reinforces that antibody testing and C-peptide evaluation should be performed in adults with atypical diabetes presentations, as long-term prospective studies have demonstrated that the presence of islet antibodies strongly predicts progressive β-cell failure [16]. Early insulin therapy, structured lifestyle support, and long-term monitoring remain the pillars of care.

## Conclusions

LADA is a heterogeneous, slowly progressive form of autoimmune diabetes that is often misclassified as T2DM. This case illustrates the importance of considering LADA in young, lean adults with worsening hyperglycemia despite oral therapy. Autoantibody testing and C-peptide assessment are critical for accurate diagnosis. This patient benefited from timely recognition and transition to insulin therapy, achieving satisfactory glycemic control over 18 months. Early insulin initiation may slow β-cell decline, reduce complications, and improve quality of life. Greater awareness of LADA among physicians is essential to avoid misclassification and delays in treatment.
